# Sensory Rewiring in an Echolocator: Genome-Wide Modification of Retinogenic and Auditory Genes in the Bat *Myotis davidii*

**DOI:** 10.1534/g3.114.011262

**Published:** 2014-08-04

**Authors:** Nicholas J. Hudson, Michelle L. Baker, Nathan S. Hart, James W. Wynne, Quan Gu, Zhiyong Huang, Guojie Zhang, Aaron B. Ingham, Linfa Wang, Antonio Reverter

**Affiliations:** * Computational and Systems Biology, CSIRO Agriculture Flagship, Queensland Bioscience Precinct, Brisbane, Queensland, Australia; † Australian Animal Health Laboratory, CSIRO Biosecurity Flagship, Geelong, Victoria, Australia; ‡ School of Animal Biology and the Oceans Institute, University of Western Australia, Crawley, WA 6009, Western Australia, Australia; § College of Information Sciences and Technology, Donghua University, Shanghai 201620, China; ** Faculty of Medicine, Imperial College London, South Kensington, London SW7 2AZ, England; †† BGI-Shenzhen, Shenzhen, 518083, China; ‡‡ Program in Emerging Infectious Diseases, Duke-NUS Graduate Medical School, Singapore

**Keywords:** evolution, vision, codon usage bias, pseudogene, bat

## Abstract

Bats comprise 20% of all mammalian species and display a number of characteristics, including true flight, echolocation, and a heightened ability to resist viral load that uniquely position this group for comparative genomic studies. Here we searched for evidence of genomic variation consistent with sensory rewiring through bat evolution. We focused on two species with divergent sensory preferences. *Myotis davidii* is a bat species that echolocates and possesses dim- but not daylight-adapted vision whereas the black flying fox (*Pteropus alecto)* has highly developed day vision but does not echolocate. Using the naked mole rat as a reference, we found five functional genes (*CYP1A2*, *RBP3*, *GUCY2F*, *CRYBB1*, and *GRK7*) encoding visual proteins that have degenerated into pseudogenes in *M. davidii* but not *P. alecto*. In a second approach genome-wide codon usage bias (CUB) was compared between the two bat species. This CUB ranking systematically enriched for vision-related (*CLN8*, *RD3*, *IKZF1*, *LAMC3*, *CRX*, *SOX8*, *VAX2*, *HPS1*, *RHO*, *PRPH2*, and *SOX9*) and hearing-related (*TPRN*, *TMIE*, *SLC52A3*, *OTOF*, *WFS1*, *SOD1*, *TBX18*, *MAP1A*, *OTOS*, *GPX1*, and *USH1G*) machinery in *M. davidii* but not *P. alecto*. All vision and hearing genes selectively enriched in *M. davidii* for which orthologs could be identified also were more biased in the echolocating *M. lucifugus* than the nonecholocating *P. vampyrus*. We suggest that the existence of codon bias in vision- and hearing-related genes in a species that has evolved echolocation implies CUB is part of evolution’s toolkit to rewire sensory systems. We propose that the two genetic changes (pseudogene formation and CUB) collectively paint a picture of that incorporates a combination of destruction and gain-of-function. Together, they help explain how natural selection has reduced physiological costs associated with the development of a smaller eye poorly adapted to day vision but that also contribute to enhanced dim light vision and the hearing adaptations consonant with echolocation.

As pointed out previously (Jones *et al.* 2013), bats perceive the world using a range of sensory mechanisms, some of which have undergone specialization (Altringham and Fenton 2003). *Myotis davidii* and *Pteropus alecto* are Chiropterans that use different habitats, prefer different diets, and occupy different sensory niches (Zhang *et al.* 2013). Previous work has compared and contrasted their genomes to shed light on the genetic basis on a host of their phenotypic similarities and differences. Recently, a new connection was drawn between bat immunity and bioenergetic demands based on gene family expansions and contractions and other lines of genetic evidence (Zhang *et al.* 2013). In taking a sensory perspective of the two species, *P. alecto* forages through vision and smell but cannot echolocate. In contrast, *M. davidii* is an echolocator thought to have relatively poor day vision. It exhibits the loss of function often observed when biological systems avoid paying the cost of unnecessary capacity (Weibel *et al.* 1991).

Basic morphological data in echolocating bats—such as differences in the lens, cornea, vitreous body shape and proportion of rods and cones—are consistent with reduced visual acuity (Bhatnagar 1975; Pettigrew 1986; Fuzessery *et al.* 1993; Kalko *et al.* 1996). A complication to the sensory ecology of myotid bats is that although they possess a smaller eye, this does not exclusively imply degradation. Recent work in *M. brandtii* suggests this echolocator may be adapted to dim light conditions such as found at dawn and dusk and also ultraviolet light (Seim *et al.* 2013). These visual adaptations are related to photopigment opsins, rhodopsin, and downstream signaling components (Seim *et al.* 2013). The molecular basis of visual adaptation in *M. davidii per se* is unknown.

Taking a broader sensory perspective, a very recently published analysis based on the ratio of nonsynonymous to synonymous substitutions common to both echolocating dolphins and echolocating bats identified genes encoding sensory proteins, particularly vision and hearing, as striking signatures of convergent evolution (Parker *et al.* 2013). The hearing adaptation relates to the echolocation facility, a multimodal capability comprising both specialized vocalization on the one hand and signal reception on the other. A recent review of the genetic basis of the sensory ecology of echolocating bats can be found here in Jones *et al.* (2013). Genes thought to be of particular interest for echolocation *per se* include those encoding proteins related to vocalization (*FOXP2*) and enhanced hearing (*SLC26A5*).

There is some debate over whether bat echolocation is (1) a derived characteristic not present in the common ancestor that has subsequently evolved in multiple echolocating lineages by convergent evolution, or (2) was present in the ancestral species then selectively lost (Teeling 2009). Either way, our aim is to contribute to the elucidation of the genetic basis of sensory rewiring, with a focus on these two species. We compare the output to two other bat species for whom genome-wide data already exist, the echolocator *M. lucifugus* and the nonecholocator *P. vampyrus*. Pteropodids diverged from other bats 55 mya, whereas the myotid bats are considered by some authorities to be a more recent lineage that split 15 mya. Assembled genomes for these four bats provide great scope for comparative genomics. Indeed, it has been pointed out that the future for understanding the molecular basis of sensory biology is promising, with comparative genomics having great potential (Jones *et al.* 2013). Here, we find a combination of hypothesis-driven and hypothesis-free approaches to be mutually reinforcing, shedding molecular insight into the apparent rewiring of *M. davidii*’s visual modality, and also complementary changes in the hearing modality that we suggest likely relate to echolocation.

Our motivation for exploring genome-wide patterns of codon usage bias (CUB) in the context of comparative mammalian evolution was our recent finding that pathway-level alterations in translation efficiency appear to have an underappreciated role in driving evolutionary change through gain of function, even in complex eukaryotes such as mammals and birds (Hudson *et al.* 2011). We previously observed systematic CUB in genes fundamental to important characteristics of each lineage, such as cell wall and chloroplast physiology in plants, mitochondrial function in birds, and hair formation in mammals (Hudson *et al.* 2011). Other recent work also has reinforced a likely role for CUB in human evolution. For example, a large scale tissue-specific analysis in humans previously found that the tRNA adapatation index significantly correlated with expression levels both globally and in a range of tissues taken in isolation (Waldman *et al.* 2010). These lines of evidence support a role for CUB in mammalian evolution, but genome-wide CUB analysis has never previously been applied to any bat species.

Here, we apply a very sensitive CUB detection tool that, when applied genome-wide, finds a coordinated pattern of enrichment for genes encoding vision and hearing-related machinery in the echolocating *M. davidii* but not the nonechlocating fruitbat *P. alecto*. Orthologs of those vision and hearing genes also were more biased in *M. lucifugus* than *P. vampyrus*, although the effect was not so strong. The outlier visual proteins tend to be highly expressed in human eye tissue but this is not always the case, implying no simple relationship to abundance. Also based on comparative human data, some of these genes are expected to have highly restricted tissue-specific expression patterns, whereas others are widely expressed. Given observations we have made applying the same method in a number of other genomes, we submit that the most likely explanation to be selection on translation efficiency that manifests at the pathway level. We propose that the coordinate impact of these changes (pseudogenes and CUB) is sensory rewiring, presumably helping to manipulate the *Myotis* retinogenic pathway toward the development of a smaller, low light-adapted eye with additional changes relating to the enhanced hearing that contributes to the echolocation facility.

## Materials and Methods

### Hypothesis-driven pseudogene analysis

A list of vision-related genes obtained from the gene ontology database (GO:0007601: visual perception) and visual system from the QIAGEN pathway library (http://www.qiagen.com/Products/Genes%20and%20Pathways/) defined presence, copy number, and nature of corresponding genes in the *M. davidii* and *P. alecto* genomes. Proteins from human vision-related genes and their neighboring genes were downloaded from Ensembl release 64. The longest transcript was chosen to represent each gene in cases when alternative splicing variants have been shown to exist. We then subjected all proteins to tBlastn analysis against the bat genomes with the similarity cutoff threshold of e-value = 1e^−5^. With the human protein set as a reference, we found the best hit for each human protein in the two bat genomes by using the criteria that more than 30% of the aligned sequence showed an identity above 30%. We used GeneWise algorithm (Birney *et al.* 2004) (with parameters -genesf -for -quiet) to define the detailed exon-intron structure of each bat gene and to identify potential pseudogenes among the bat visual genes.

Initially, we targeted the examination of the bat orthologs for vision-related pseudogenes identified in the naked mole rat *Heterocephalus glaber* (Kim *et al.* 2011), a species with poor vision adapted to life in the dark. Genes containing frame shifts or premature stop codons were considered candidates. We filtered as follows: (1) To avoid reported frame shifts or premature stop codons that were due to a flaw in the GeneWise algorithm, we aligned all human proteins to their corresponding loci in the human genome, and genes with frame shifts or premature stop codons in human-to-human alignments were filtered; (2) Using the results of the human-to-human alignment from GeneWise, candidate pseudogenes with obvious splicing errors near their frame shifts or premature stop codons were filtered; (3) Candidate pseudogenes with a low number of reads covering their frame shift or premature stop codon sites were considered assembly errors. Genes with a considerable number of reads due to genotype variation at these sites were treated as heterozygous and filtered.

### Hypothesis-free genome-wide CUB analysis

A number of CUB statistics have been proposed, and there is little consensus on the optimal approach. A very recent review outlining the pros and cons of some commonly applied CUB quantification approaches can be found here in Behura and Severson (2013). Our CUB statistic uses the concept of entropy, which estimates data regularities based on a combination of order and proportion. Its strength lies in its broad applicability and sensitivity. Because it is not systematically influenced by gene length and accounts for amino acid composition, it can be fairly applied within and between genomes. It does not account, however, for background GC content, nor does it determine whether bias is toward preferred or nonpreferred codons. For this reason, background GC content and a more detailed analysis of codon usage must be retrospectively assessed on a case-by-case basis (*i.e.*, in those genes whose entropic properties have been determined as extreme and therefore worthy of deeper investigation).

To summarize, we determined the extent of CUB for every coding sequence using an information theory-based statistic as previously described (Hudson *et al.* 2011). For each observed coding sequence, we computed the entropy of the nucleic acid and compared it against the average entropy of 20 random sequences. The random sequences were generated in a way that they would encode the same amino acid sequence, but where the codons were selected at random. Because entropy provides a measure of data regularity, the differential entropy between the observed sequence and the random corresponds to the extent of regularity attributable to CUB.

We first ranked all coding sequences on extent of CUB on a within-genome basis. To ascertain the extent of functional enrichment analysis the ranked lists for *Myotis* and *Pteropus* were subjected to the GOrilla webtool (Eden *et al.* 2009). GOrilla uses hypergeometric statistics to determine whether particular biological processes are enriched within any ranked input set of genes. We used the single ranked list option based on human functional annotation, and imported SWISSPROT IDs, which is one of the designated preferred input identifiers for GOrilla analysis. We also compared relative codon bias in the orthologs in common between the two species (of which we identified 6748). This secondary analysis helps identify genes that may not be extreme on a within genome basis but still possess markedly different properties between the two species.

Because the GOrilla enrichment relies on incomplete annotation of functional processes and can overlook functional connections even in well-annotated genes, we used the top enrichment as a focus for further manual curation, based on mining the PubMed literature database. A subset of outlier CUB genes were retrospectively assessed for regional GC content. This was computed from the gene itself, plus 10 kb upstream from the start codon and 10 kb downstream from the stop codon.

#### Independent validation of divergent CUB:

The differential entropy statistic is one of many ways of measuring CUB. In an effort to provide an independent line of evidence for differential CUB in the two bat species and subsequent differential functional enrichment, we also ran the previously published effective numbers of codons (ENC) (Wright 1990) through the CodonW website: http://codonw.sourceforge.net/. According to Behura and Severson (2013), ENC is used to measure how far the codon usage of a gene departs from equal usage of synonymous codons. EC values range from 20 (extreme codon bias when one codon is used exclusively for each amino acid) to 61 (in cases of no codon bias where synonymous codons are equally likely to code the amino acid).

#### CUB outlier genes quality checking:

Because it is possible that gaps in the genome assembly data can result in artifacts with the cds sequences, the cds sequence data for all *M. davidii* CUB outlier genes were confirmed by comparison with cds sequences from other species. We used translation BLAST to identify potential assembly problems, including gaps in our cds sequences. The bats *M. brandtii* and *M. lucifugus* plus other mammalian species available in Genbank were used for sequence alignment. In the event of potential missing exons we cross-referenced back to the *M. davidii* genomic assembly to confirm the quality of genomic sequence data.

#### CUB analysis in two additional bat species: M. lucifugus and P. vampyrus:

To detect comparative evidence for CUB in the sensory apparatus in echolocating bats, we computed CUB in an additional echolocator (*M. lucifugus*) and nonecholocator (*P. vampyrus*). The current genome assemblies from the Ensemble database for *P. vampyrus* (pteVam1) and *M. lucifugus* (Myoluc2.0) were used for this analysis. For these two comparison species, we obtained coding sequence data from Biomart, yielding 14,141 annotated cds for *M. lucifugus* and 8, 845 for *P. vampyrus*. CUB was estimated using the entropy statistic as previously described.

## Results

### Hypothesis-driven pseudogene analysis

**Figure 1:**
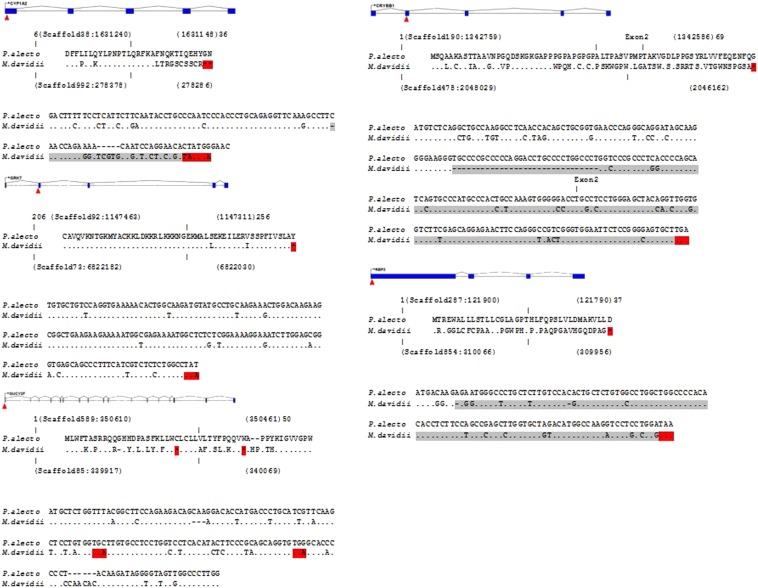
Gene structure, partial amino acid ,and nucleotide alignments for five *M. davidii* pseudogenes. Red triangles illustrate the location of stop codons compared with *P. alecto*. Amino acid alignment surrounding the stop codon region in *P. alecto* and *M. davidii*. Asterisks represent stop codons. Nucleotide alignment between *P. alecto* and *M. davidii* corresponding to the same region in amino acid alignment. Gray nucleotides represent the region after a frame shift in *M. davidii* that leads to a premature stop codon (*red*).

Of the 19 visual perception genes identified as inactivated or missing in the naked mole rat, we identified five that have also become pseudogenes in *M. davidii* but not *P. alecto (CYP1A2*, *RBP3*, *GUCY2F*, *CRYBB1*, and *GRK7*; Figure 1). Taxonomically the naked mole rat *H. glaber* is placed within rodents. Despite some exceptional traits the overall properties of the *H. glaber* genome are similar to other mammals (Kim *et al.* 2011) making the comparison to the two bat species reasonable.

For *CYP1A2*, *CRYBB1*, and *RBP3* single or multiple nucleotide deletions were responsible for frame-shift mutations leading to premature stop codons in *M. davidii*. For *CRYBB1*, the deletion was 31 nucleotides in the first exon compared with *P. alecto*. In contrast, the *GRK7* and *GUCY2F* pseudogenes were caused by single-point mutations that created single stop codons. In the case of *GUCY2F* two point mutations had created two stop codons in close vicinity to each other. The similarity between the intact *P. alecto* genes compared to their corresponding regions in the *M. davidii* genome, ranged from 85 to 90%.

### Hypothesis-free genome-wide CUB analysis

We calculated codon usage preferences for the two species genome-wide (Table 2 and Table 3). These genome-wide figures can be used to help establish preferred and nonpreferred codons for that particular species in concert with the available tRNA pool. In turn this helps shed light on the translational implications of CUB. Namely, is CUB likely to increase or decrease the energetic cost and efficiency of translation for the gene in question?

We next used a hypothesis-free screen, based on CUB, to test for pathway functional enrichment. Before functional enrichment, GOrilla screens the imported gene list, removes duplicates (keeping the only the most highly ranked candidate), then assigns GO characteristics where those exist. For *M. davidii* we imported 13,654 genes that could be assigned SWISS PROT identifiers, of which 37% were recognized. A total of 6951 were recognized by Gene Symbol, 1731 duplicates were removed, and of the remaining 5220, 5056 had a GO term assigned. For *P. alecto* we imported 12,188 genes that could be assigned to SWISS PROT identifiers of which 40% were recognized. A total of 6183 were recognized by Gene Symbol, 1056 duplicates were removed, and of the remaining 5127, 4956 had a GO term assigned.

*NEFH* (alias *YHU2*, encoding the neurofilament heavy polypeptide) possessed extreme CUB in both species. Consistent with previous research in other organisms, the translation initiation factor *IF2* (*PRP2*) was among the most biased genes in both species.

**Figure 2:**
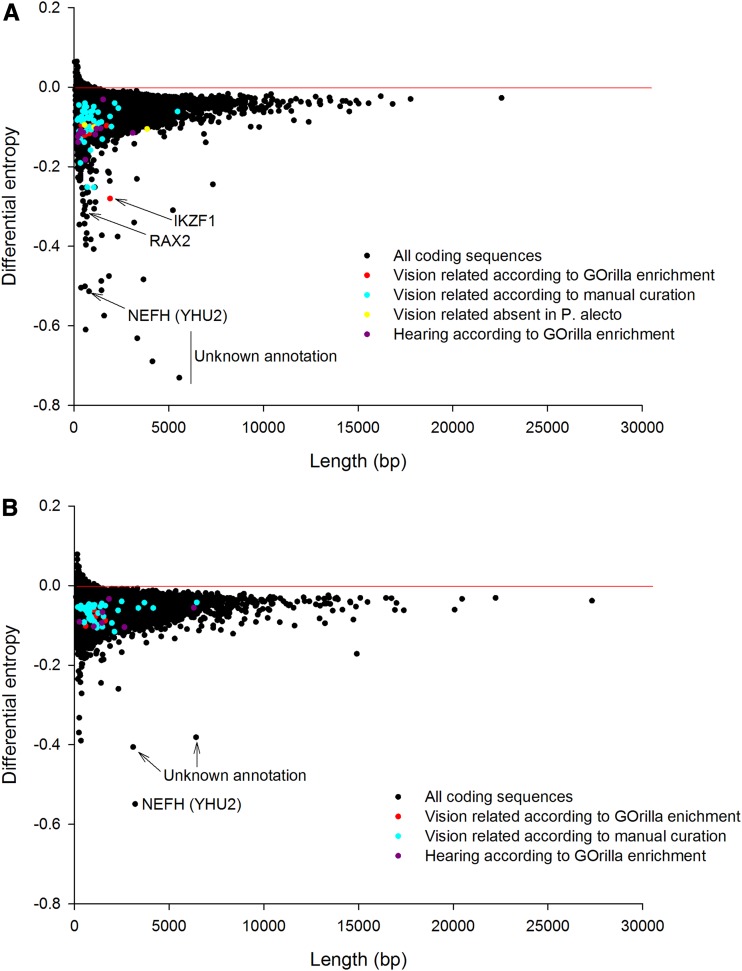
Genes showing extreme codon bias (negative differential entropy) on a within-genome basis in (A) *M. davidii* and (B) *P. alecto*, with those encoding vision and hearing related proteins highlighted.

Of the biased genes within *M. davidii* we found the top ‘Process’ enrichment to be “*retina development of camera type eye*” (hypergeometric test *P*-value = 0.0000775; False Discovery Rate q-value = 0.766) based on the prominent bias of the following genes: *CLN8*, *RD3*, *IKZF1*, *LAMC3*, *CRX*, *SOX8*, *VAX2*, *HPS1*, *RHO*, *PRPH2*, *SOX9*; Figure 2A and Supporting Information, File S1). The remaining functional enrichments reported in descending order by GOrilla for *M. davidii* were “*O-glycan processing*” (*P*-value = 0.000127), “*protein O-linked glycosylation*” (*P*-value = 0.000393), “*adenylate cyclase-inhibiting G-protein coupled receptor signalling pathway*” (*P*-value = 0.000508), “*renal absorption*” (*P*-value = 0.00051), “*renal water transport*” (*P*-value = 0.00051), “*peripheral nervous system neuron axonogenesis*” (*P*-value = 0.000593), “*positive regulation of transcription from RNA polymerase II promoter in response to stress*” (*P*-value = 0.000791), “*sensory perception of light stimulus*” (*P*-value = 0.000853), “*sensory perception of sound*” (*P*-value = 0.000932), and “*negative regulation of neutrophil differentiation*” (*P*-value = 0.000989).

The ‘*sensory perception of light stimulus*’ contained the following additional genes not recognized in the ‘*retina development*’ function, but which play a related physiological role: *OPN4*, *NR2E3*, *CNGB1*, *RDH8*, *ROM1*, *CLN6*, *WFS1*, *KIFC3*, *RGR*, *RP1L1*, *RDH5*, *CABP4*, *AIPL1*, *RAX2*, *NRL and USH1G*. The sensory perception of sound comprised the following genes (*TPRN*, *TMIE*, *SLC52A3*, *OTOF*, *WFS1*, *SOD1*, *TBX18*, *MAP1A*, *OTOS*, *GPX1* and *USH1G*). Overall, we considered the collective presence of 4 functions in *M. davidii* (retina development, peripheral axonogenesis, light perception and sound perception) clearly associated with sensory rewiring to be noteworthy.

There was no formal vision related enrichment detected for *P. alecto* (Figure 2B) or indeed of any other sensory rewiring such as sound perception. The top ‘Biological Process’ enrichments in descending order were “*cellular developmental process*” (*P*-value = 0.0000178), “*regulation of intrinsic apoptotic signalling pathway*” (*P*-value = 0.000559), “*G-protein coupled receptor signalling pathway*” (*P*-value = 0.000634), “*developmental process*” (*P*-value = 0.000192), “*regulation of interferon-gamma biosynthetic process***”** (*P*-value = 0.000267), “*positive regulation of MAPK cascade*” (*P*-value = 0.000354), “*cell differentiation*” (*P*-value = 0.000381), “*cell surface receptor signalling pathway*” (*P*-value = 0.00056),“*regulation of MAPK cascade*” (*P*-value = 0.000652), “*regulation of interferon-gamma production*” (*P*-value = 0.000703) and “*blood vessel development*” (*P*-value = 0.000813). In both species, there was some indication of enrichment relating to immune function.

**Figure 3:**
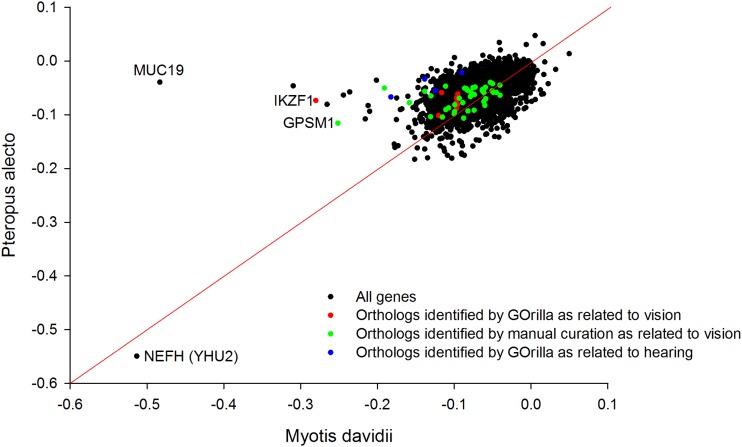
Orthologs showing differential codon bias (distance from diagonal) between *M. davidii* and *P. alecto* on a between-genome basis. Genes encoding vision and hearing elated proteins are highlighted. Of the sensory genes *IKZF1* is particularly prominent. The highly codon biased *LAMC3*, *RHO*, and *SOX9* identified by GOrilla as vision-related in *M. davidii* appear to be absent in *P. alecto*. *MUC19*, highly biased in *M. davidii*, is a Mucin family member expressed in the inner ear. *NEFH* is equally highly biased in both bat species.

When we compared these orthologs of the two genomes directly on a between-genome basis (Figure 3), *IKZF1* (also identified in the “retina development of the camera eye”) displayed a prominent outlier value in *M. davidii*. *LAMC3*, *RHO*, and *SOX9* were genes annotated as vision related that were absent in the *P. alecto* annotation and so orthologs could not be directly compared. A number of other orthologs were found to possess more bias in Myotis than in Pteropus, some of which were manually explored for their possible connection to the visual system. Although not identified as relating to hearing by GOrilla enrichment, the outlier position of *MUC19* is of interest, given (1) the general role of mucin family members in signal transduction during hearing (Kerschner 2007) and (2) that *MUC19* specifically encodes a major gel-forming mucin expressed in the middle ear (Kerschner *et al.* 2009). However, the mucins are known to bind pathogens, so an immune function or some other role cannot be discounted.

The overall distribution of these data was not symmetrical. There were more relatively biased orthologs in *M. davidii* than in *P. alecto*. This may reflect the fact that the Myotis bats are a much more recent lineage that, according to some sources, diverged approximately 15 mya from other bat species. Alternatively, the skewed data may reflect the fact that *M. davidii* possesses more derived phenotypic characteristics than *P. alecto*, resulting from the evolution of enhanced echolocation in this species. The genome-wide GC content is similar in the two bat species, with *P. alecto* (*39.7%*) slightly more biased than *M. davidii* (42.7%). This finding implies genome-wide GC content does not account for the slight skew in the between species distribution.

It is clear from scrutinizing the plots that the CUB statistic has the appealing feature of being independent of gene length because the mass of the data in Figure 1 follows a horizontal line in both species. It is also immediately apparent that, sitting close to the horizontal line, the vast majority of genes in both bat species do not exhibit extreme CUB. Nevertheless, in both cases there are a small subset of genes, many of which are short and some of which are long, that possess properties of relatively extreme CUB. Moreover, this subset enriches for a particular biological function, even though the physical location, gene length, and other molecular details vary. This observation can be generalized. We detected the exact same broad patterns previously identified in six other eukaryotic genomes: Arabidopsis, *S. cerevisiae*, *C. elegans*, chicken, chimpanzee, and human (Hudson *et al.* 2011) *i.e.*, whereas most genes are unbiased, a small subset of variable length are biased and these enrich for particular functions that appear relevant to that lineage.

We calculated the GC content up and downstream of the differentially biased *IKZF1*, *GPSM1*, and *SPRR1* (cornifin) and found them to be either almost identical, or more out of equilibrium in the Pteropodid genome. Table 1 summarizes these findings. Therefore, regional background GC content is unable to account for the greater CUB in these Myotid coding sequences. Gene length changes between species is also unable to account for the observation of differential CUB, as although it varies, the variation is not systematic. *M. davidii IKZF1* is longer than in *P. alecto*, *GPSM1* is shorter, and *SPRR1* is almost identical, whereas CUB is substantially more extreme in *M. davidii* in all three cases.

**Table 1:** The genomic properties of three vision-related genes showing more CUB in *M. davidii* than *P. alecto*

Gene	GC% *M. davidii*	GC% *P. alecto*	Gene length *M. davidii*	Gene length *P. alecto*
*IKZF1*	49.6	53.2 / 51.5	1908	228/816
*GPSM1*	49.1	65.4	705	2118
*SPRR1*	44.0	47.1	339	342

Background GC% and gene length cannot account for the observation. CUB, codon usage bias.

#### Independent validation of divergent CUB:

The ENC measure of genome-wide CUB reinforced the change in the visual system of *M. davidii* and partially reinforced the change in its auditory system compared with *P. alecto*. However, the sensory enrichments observed for *M. davidii* were no longer ranked among the top ten on a within-genome basis.

The specific functional enrichments “retina development in camera-type eye” (based on *CLN8*, *BAX*, *PRPH2*, *LAMC3*, *IKZF1*, *SOX9*, *NPHP4*, *PFDN5*, *PVRL1*, *SOX8*, *RD3*) and “sensory perception of sound” (based on *ASIC2*, *TPRN*, *MYO15A*, *WFS1*, *SOBP*, *ESPN*, *GPX1*, *TIMM13*, *GRM7*, *TMIE*, *SLC52A3*, *OTOF*, *SOD1*, *MAP1A*, *OTOS*, *SIX1*, *USH1G*, *MYO7A*) were both significant in *M. davidii* (*P* = 0.000294 and *P* = 0.00066, respectively) but not *P. alecto*, broadly reinforcing the patterns identified by the original analysis performed using the entropy-based statistic. Interestingly, the Myotid bat also had the functional enrichment (*P* = 0.000232) “sensory perception of mechanical stimulus” (based on *ASIC2*, *TPRN*, *MYO15A*, *TSHZ3*, *WFS1*, *SOBP*, *GPX1*, *TIMM13*, *GRM7*, *TMIE*, *SLC52A3*, *OTOF*, *SOD1*, *MAP1A*, *OTOS*, *SIX1*, *USH1G*, *MYO7A*). The overlap in content with “sensory perception of sound” given genes such as *TMIE* (*trans*-membrane of inner ear), and components of the otolith (*OTOF* and *OTOS*) suggests this function is also essentially related to inner ear development.

Vision enrichments for *P. alecto* are present but to a lesser extent and do not convincingly highlight sensory rewiring. Although *P. alecto* does have vision-related enrichments given the presence of “closure of optic fissure” (*P* = 0.00026) “positive regulation of lens epithelial cell proliferation” (*P* = 0.00026), and “regulation of lens epithelial cell proliferation” (*P* = 0.00026), these were all based on the ranked position of a single gene, *SOX11*. Finally, “Inner ear development” (based on *PDGFA*, *CCM2*, *CYTL1*, *HPCA*, *LHX3*, *GATA3*, *SHH*, *CEBPA*, *SOX2*, and *SIX1*) was enriched in Pteropus (*P* = 0.000816). The genes comprising the hearing enrichment in *P. alecto* are different to those highlighted in *M. davidii*.

**Figure 4:**
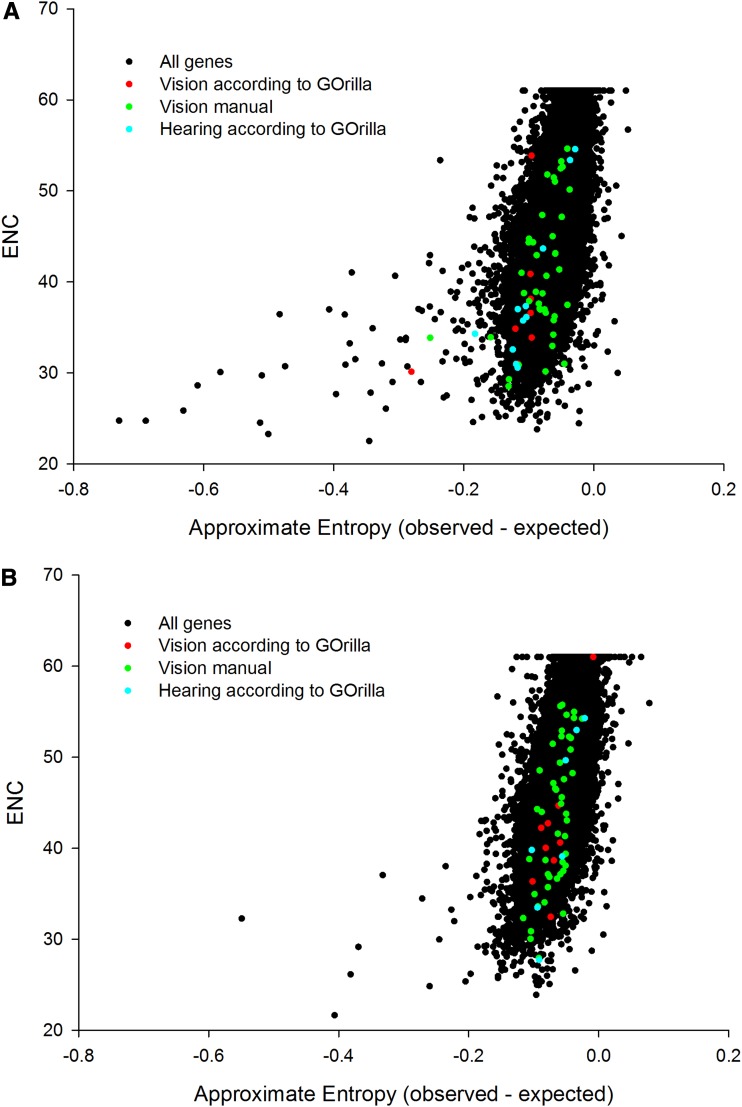
The relationship between ENC and entropy based CUB estimation for (A) *M. davidii* and (B) *P. alecto*.

The genome-wide relationship between the two different CUB statistics (entropy *vs.* ENC) was plotted (Figure 4). We discovered a positive relationship (correlations of 0.619 and 0.672 in *M. davidii* and *P. alecto*, respectively). The entropy CUB statistic appears to discriminate the highly biased genes more sensitively, as evidenced by the skew at the bottom left of both species. This enhanced discrimination of entropy as one assesses CUB in the more biased genes appears a possible driver of any observed discrepancies in functional enrichment.

#### CUB outlier genes quality checking:

*M. davidii LAMC3*, *CLN8*, and *SOX9* contained apparent gaps in the cds after translation BLAST and comparison with other bats and mammals. *IKZF1*, *WFS1*, and *SOX8* were in the expected reading frame but not contiguous. Collectively, these genes possess low-quality genomic sequence (string of Ns) in predicted exonic regions that might explain any missing cds sequence compared with other mammals. In contrast, alignment of *M. lucifugus* sequence for *IKZF1*, *LAMC3*, *SOX9*, and *SOX8* to other mammals is more conserved. This finding implies these genes may contain genomic assembly errors peculiar to *M. davidii* rather than being indicative of true evolutionary change. *SOD1* appears to be partial sequence only.

#### CUB enrichment across two additional bat species: M. lucifugus and P. vampyrus:

**Figure 5:**
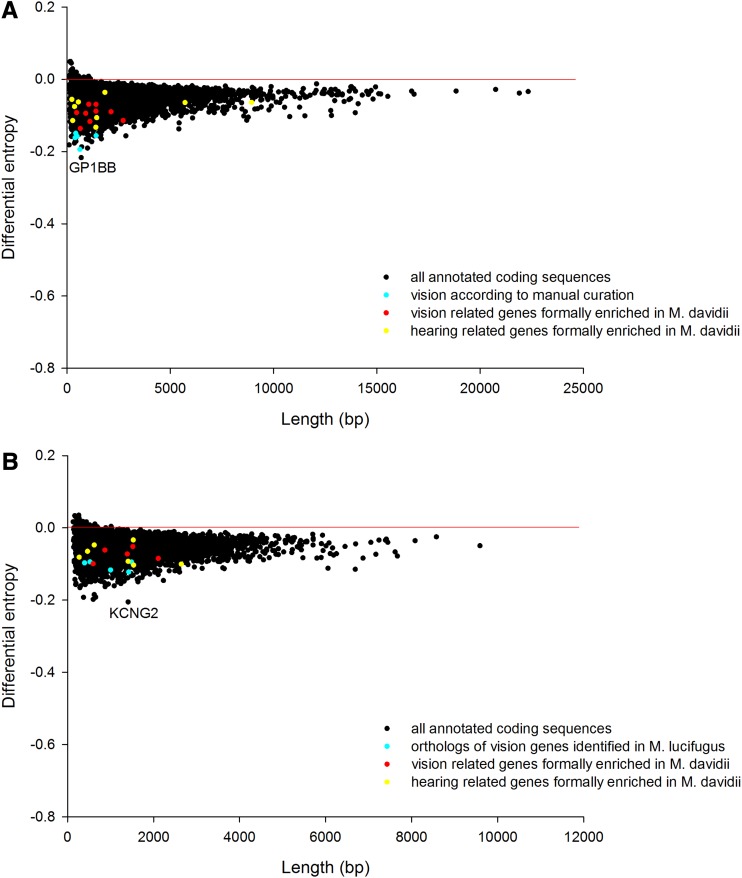
Genes showing extreme codon bias (negative differential entropy) on a within-genome basis in (A) *M. lucifugus* and (B) *P. vampyrus*, with those encoding vision and hearing related proteins highlighted.

In an effort to find independent comparative evidence for CUB in genes encoding proteins related to the sensory apparatus of echolocating bats, we computed the entropy based CUB in an additional echolocator (*M. lucifugus*) and a nonecholocator (*P. vampyrus*) species (File S2 and Figure 5). Sensory perception was not enriched in either species according to GOrilla analysis. The top three functional enrichments for *M. lucifugus* were G-protein−coupled receptor signaling pathway (*P* = 0.000000137), G-protein−coupled receptor signaling pathway coupled to cyclic nucleotide messenger (*P* = 0.00000667), and negative regulation of blood pressure (*P* = 0.0000295). For *P. vampyrus* they were G-protein−coupled receptor signaling pathway (*P* = 0.0000000196), adenylate cyclize-activating G-protein−coupled receptor signaling pathway (*P* = 0.0000296), and feeding behavior (*P* = 0.0000315).

**Figure 6:**
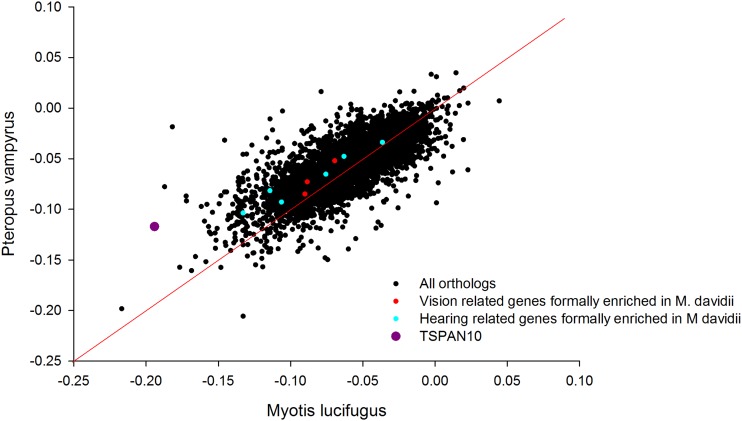
Orthologs showing differential codon bias (distance from diagonal) between *M. lucifugus* and *P. vampyrus* on a between-genome basis. Genes encoding vision and hearing elated proteins are highlighted. Of the sensory genes, *TSPAN10*, which has a highly restricted expression pattern in retina, is particularly prominent. All the sensory genes prioritized by CUB in *M. davidii* are more biased in *M. lucifugus* than *P. alecto*.

Next, we identified the orthologs of those vision and hearing sensory genes previously enriched in *M. davidii*. In the three vision (*SOX8*, *HPS1*, *IKZF1*) and six hearing (*TMIE*, *SLC52A3*, *TBX18*, *OTOS*, *GPX1* and *USH1G*) instances in which both *M. lucifugus and P. vampyrus* orthologs could be identified, they were always more biased in *M. lucifugus* than *P. vampyrus* (Figure 6).

Finally, we manually explored the *M. lucifugus* and *P. vampyrus* CUB outliers by using the GO information in the Gene function of the National Center for Biotechnology Information database and searching the literature in PubMed. In *M. lucifugus* on a within-genome basis the second (*TSPAN10*, alias *OCSP*) (Wistow *et al.* 2002), 15th (*HCRT*) (Liu *et al.* 2011), 20th (*FIZ1*) (Mali *et al.* 2008), 25th (*RTN4R*) (Wang *et al.* 2008), and 34th (*TRAPPC6A*) (Gwynn *et al.* 2006) were all vision-related (Figure 5A). *TSPAN10* has a very strong tissue-restricted expression in retina based on the BioGPS normalized tissue expression library (Wu *et al.* 2009). In fact, retina is the only tissue of the 84 assayed in which *TSPAN10* has an expression level greater than threefold of the mean (File S3). *FIZ1* is a transcriptional co-factor that interacts with *NRL* and *CRX* to drive expression of rod photoreceptor-specific genes (Mali *et al.* 2008). Intriguingly, one of these partners (*CRX*) was actually prioritized by CUB in *M. davidii*. *TRAPPC6A* contributes to melanosome formation, and when mutated in mouse yields a loss of pigmentation phenotype in the retinal epithelial layer (Gwynn *et al.* 2006).

Although there was no formal enrichment for sensory perception in *M. lucifugus*, the CUB data obtained for the two myotid bats is broadly comparable at a pathway level (vision and hearing). Although some details differ, this is highly suggestive of a role for CUB in echolocating bat sensory evolution.

## Discussion

In this study we performed a comparative genomic assessment of Myotid and Pteropodid bats by using multiple approaches, and the results provide information on the genetic origins of sensory rewiring in an echolocating bat. We began by identifying pseudogenes using the naked mole rat as a comparative species with poor vision. Pseudogenes are relatives of genes that have subsequently lost their protein coding function, usually by disabling mutations such as premature stop codons and frameshifts. The observed correspondence between the naked mole rat and the echolocating bat (*CYP1A2*, *RBP3*, *GUCY2F*, *CRYBB1*, and *GRK7*) points to the general importance of these particular genes in controlling mammalian vision and also to pseudogenes in particular as a genetic signature after loss of physiological function (Newman and Robinson 2005; Meredith *et al.* 2013).

The next analysis was a hypothesis-free screen based on the genome-wide extent of CUB. In simple organisms, CUB is known to alter translation efficiency and subsequent protein abundance, but the potential role in governing translation efficiency during mammalian evolution is less clear (Duret 2002). Supporting the concept it is known that protein expressed from a foreign gene in human cells can be increased by two orders of magnitude if the sequence is engineered to contain the most commonly used human codons (Gustafsson *et al.* 2004). However, there is an apparent lack of correlation between codon bias and protein abundance, and it is possible for codon-usage studies to be systematically influenced by amino acid content, gene length, and other potential artifacts (Chamary *et al.* 2006).

Despite these variable findings, all these studies have been impacted by an inability to exhaustively interrogate an entire and well-annotated genome (Behura and Severson 2013). For this reason, recent work that focuses on complete genome screens as well as other factors such as accounting for tissue and developmental stage, along with this study, may provide the first complete evidence for correlation between CUB and protein abundance in humans and other mammals (Waldman *et al.* 2010). A recent study concluded that although variation in mutational bias is the dominant force influencing CUB, translational selection acts as a weak additional factor and that this is widely true across the vertebrates (Doherty and McInerney 2013).

On a within-genome basis, we were struck with the finding that the top functional enrichment for the echolocating *M. davidii* was retina development, a process not enriched at all on a within-genome basis in *P. alecto*. The additional *M. davidii* enrichments for ‘*axonogenesis*’, ‘*light perception*,’ and ‘*sound perception*’ that were absent in *P. alecto* reinforced evidence for sensory rewiring in the echolocating species. Equivalent analyses run on the additional echolocator *M. lucifugus* and the additional nonecholocator *P. vampyrus* did not receive formal enrichment scores for vision and hearing. However, manual exploration of the outliers found intriguing support for vision, particularly through *TSPAN10*, which has a very prominent tissue specific expression in retina, and the transcriptional co-activator *FIZ1*, which interacts with *CRX* to drive the expression of rod photoreceptor-specific genes.

To complement our CUB within-genome analysis (Figure 2), we also compared the orthologs of *M. davidii* and *P. alecto* directly (Figure 3). Orthologs identified as substantially more biased in *M. davidii* (but not previously identified by the within-genome GOrilla analysis) were explored manually for possible connections to the visual and hearing systems through targeted PubMed literature searches. Although these genes are bound together by their shared role in the biological processes of vision and hearing, they are somewhat diverse in terms of other more detailed molecular and functional properties. In regard to the visual proteins, some are transcription factors (*e.g.*, *CRX*), whereas others are visual pigment proteins (*e.g.*, *RHO*). Based on the TIGER database (Liu *et al.* 2008), which provides normalized tissue mRNA comparisons in humans, many of the vision genes are abundantly expressed in eye, and some are not. Furthermore, some are expressed highly across a wide range of tissues whereas others have an extremely restricted expression profile.

Clearly, this range of tissue-level expression profiles renders any simple correlation of CUB to protein abundance unlikely. However, this does not mean one should discount CUB as a driver of eukaryotic evolution, as previously argued (Waldman *et al.* 2010). Waldman *et al.* (2010) found significantly greater correlations observed in adult tissues than the fetus, pointing to the importance of developmental stage in these kind of assessments (Waldman *et al.* 2010). In that work, related sets of functional gene groups were found to be translated efficiently in each tissue, pointing to the importance of viewing groups of genes as components in a broader biological process or pathway (Waldman *et al.* 2010). In the future, comparison of biased genes to actual expression level in components of the bats sensory system would be of interest, but unfortunately these data are not presently available.

To allow the CUB outliers to be placed in context, we calculated the genome-wide preferred codons for *M. davidii* and *P. alecto* based on all the available coding sequences available from the genome sequencing efforts (Table 2 and Table 3). These data can be exploited to make predictions of bias toward or against preferred codons. In the context of explaining the low light−adapted vision and small eyes of *M. davidii*, we would predict enhanced translation efficiency (bias toward preferred codons) in negative regulators of eye size and visual pigments adapted for low light, and the converse (nonpreferred codons) for positive regulators of eye size and pigments adapted for day vision. However, the developmental pathways controlling retinogenesis are incomplete, and the genetic changes in the highlighted genes need to be assessed on a case-by-case basis and await future functional validation. Of the vision related genes highly biased in *M. davidii IKZF1* encodes a transcription factor called Ikaros family zinc finger 1, an ortholog of which confers competence to mouse retinal progenitor cells (Elliott *et al.* 2008). Manual exploration identified additional highly differentially biased genes encoding proteins expressed in eye components—*GPSM1* (Kerov *et al.* 2005) and *SPRR1* (Tong *et al.* 2006). Enrichments for light and sound perception include numerous additional sensory genes not included in the specific retinogenic enrichment. The observation of extreme CUB in CRX in *M. davidii* and its binding co-factor FIZ1 in *M. lucifugus* supports this molecular pathway as a likely hotspot in vision-related adaptations that have evolved in nocturnal echolocating bats.

**Table 2:** Patterns of genome-wide codon usage in *Myotis davidii*

UUU	15.7 (141141)	UCU	14.4 (128939)	UAU	10.7 (96582)	UGU	9.7 (87355)
UUC	20.5 (183882)	UCC	19.1 (171243)	UAC	15.5 (139000)	UGC	12.6 (112906)
UUA	7.2 (64716)	UCA	11.3 (101170)	UAA	0.6 (5059)	UGA	1.2 (10439)
UUG	12.5 (112218)	UCG	5.0 (45139)	UAG	0.5 (4775)	UGG	12.4 (111457)
CUU	12.1 (108263)	CCU	16.9 (151920)	CAU	10.0 (90245)	CGU	4.2 (37777)
CUC	20.4 (183451)	CCC	21.6 (194040)	CAC	16.6 (148955)	CGC	9.9 (89098)
CUA	6.4 (57262)	CCA	15.9 (143248)	CAA	11.8 (106136)	CGA	6.0 (54257)
CUG	41.3 (371130)	CCG	7.6 (68087)	CAG	36.1 (323855)	CGG	12.0 (108187)
AUU	14.6 (131554)	ACU	12.0 (108140)	AAU	15.5 (139605)	AGU	12.1 (108595)
AUC	21.5 (193277)	ACC	19.8 (177653)	AAC	20.0 (179409)	AGC	20.5 (183722)
AUA	6.9 (62193)	ACA	13.9 (125180)	AAA	23.6 (211742)	AGA	11.5 (103418)
AUG	22.1 (198262)	ACG	7.2 (65088)	AAG	33.3 (298847)	AGG	12.9 (115789)
GUU	10.3 (92630)	GCU	17.9 (161068)	GAU	20.8 (186580)	GGU	9.9 (89712)
GUC	15.4 (137894)	GCC	29.3 (263071)	GAC	27.4 (246001)	GGC	22.2 (199804)
GUA	6.4 (57396)	GCA	15.2 (136268)	GAA	28.7 (25792)	GGA	15.5 (138999)
GUG	29.2 (261930)	GCG	7.6 (68234)	GAG	41.6 (374083)	GGG	17.3 (155758)

These data help set the expectation for whether a given *M. davidii* codon is preferred or nonpreferred. Observed bias in any particular gene can therefore be interpreted as likely to increase or decrease the cost and efficiency of translation

**Table 3:** Patterns of genome-wide codon usage in *Pteropus alecto*

UUU	16.3 (156476)	UCU	15.2 (146151)	UAU	11.3 (108424)	UGU	10.0 (96432)
UUC	20.0 (192106)	UCC	17.7 (170229)	UAC	14.8 (141890)	UGC	12.2 (116902)
UUA	7.7 (74291)	UCA	12.0 (114942)	UAA	0.5 (5244)	UGA	1.1 (10325)
UUG	13.1 (125571)	UCG	5.2 (49969)	UAG	0.5 (4808)	UGG	12.3 (117792)
CUU	13.1 (125218)	CCU	17.6 (168660)	CAU	10.4 (100155)	CGU	4.9 (46662)
CUC	19.8 (190319)	CCC	20.7 (198601)	CAC	15.7 (150619)	CGC	10.6 (101489)
CUA	7.3 (69639)	CCA	16.7 (160345)	CAA	12.2 (116897)	CGA	6.4 (61551)
CUG	39.5 (379104)	CCG	7.7 (74204)	CAG	35.0 (336173)	CGG	12.0 (114783)
AUU	15.1 (144545)	ACU	12.9 (124104)	AAU	16.3 (156140)	AGU	12.5 (119863)
AUC	20.4 (196052)	ACC	18.5 (177795)	AAC	18.9 (181664)	AGC	20.1 (192594)
AUA	7.3 (70411)	ACA	14.6 (139725)	AAA	24.3 (233289)	AGA	11.8 (112920)
AUG	21.5 (206522)	ACG	6.9 (65967)	AAG	31.9 (305590)	AGG	12.1 (116001)
GUU	10.6 (101643)	GCU	18.5 (177510)	GAU	21.4 (205693)	GGU	10.5 (100839)
GUC	15.2 (146033)	GCC	28.8 (275932)	GAC	26.3 (253066)	GGC	22.5 (215905)
GUA	7.2 (69462)	GCA	15.7 (150363)	GAA	29.6 (284341)	GGA	15.8 (151181)
GUG	27.9 (267333)	GCG	8.0 (76818)	GAG	40.3 (387415)	GGG	16.6 (159580)

These data help set the expectation for whether a given *P. alecto* codon is preferred or nonpreferred. Observed bias in any particular gene can therefore be interpreted as likely to increase or decrease the cost and efficiency of translation.

Overall, our findings across two echolocating (*M. davidii* and *M. lucifugus*) and two nonecholocating (*P. alecto* and *P. vampyrus*) bats strongly support a recent observation made across echolocating mammals (bats and bottlenose dolphins) using a different comparative genomics approach. In this independent study, it was found that patterns of convergent protein evolution (based on nonsynonymous *vs.* synonymous amino acid substitutions) identify pathways contributing to hearing and vision as among the strongest signals (Parker *et al.* 2013). The exact overlap between our differential CUB analysis *vs.* the protein evolution study of (Parker *et al.* 2013) included the genes *RHO* and *OTOF*, which are involved in light and sound perception, respectively. The fact that not all genes exist in both analyses suggests that multiple regulatory mechanisms are likely to contribute to change of function.

*NEFH* (alias *YHU2*; encoding the neurofilament heavy polypeptide) possessed extreme CUB in both *M. davidii* and *P. alecto*, implying it was either present in the ancestor or has subsequently converged due to a fundamental role in both bat lineages. A broader comparative analysis is needed to shed light on this gene, but unfortunately an annotated ortholog has not been identified in either the *M. lucifugus* or *P. vampyrus* sequences. One could sequence *NEFH* in a range of bats and one or more Laurasiatherian outgroups. *NEFH* is most highly expressed in the peripheral nervous system (Liu *et al.* 2008), so a generic sensory explanation seems plausible *i.e.*, one not specific to echolocation. Finally, a number of unannotated highly biased genes in both *M. davidii* and *P. alecto* were detected. These may represent targets for future functional studies.

Overall, our findings provide insight into two genetic mechanisms contributing to evolution’s toolkit for adapting phenotypes. The deterioration of a number of core visual genes in *M. davidii* is consistent with genomic evolution in the naked mole rat (Kim *et al.* 2011) and the poor day vision and small eyes of this species. Pseudogene formation is clearly a loss-of-function decay process. Disabling mutations are allowed to persist because of relaxation of selection pressure on that particular protein. Because mutation is random, if unchecked it will cause DNA sequence to tend toward (random) equilibrium. This finding contrasts with extreme CUB, which enforces structure and regularity on DNA sequence. This enforced regularity is strongly indicative of an applied evolutionary force, and tends to be associated with gain-of-function.

The CUB evidence we have gathered for manipulation of translation efficiency at the biological pathway level has not previously been reported in the context of bat sensory ecology and warrants further investigation. The CUB data collectively imply a suite of adaptations to both the visual system (perhaps positively impacting on adaptation to dim light) and the enhancement of hearing (which presumably relates to echolocation). Overall, the general prioritization of sensory rewiring in the echolocator is highly resonant with the recent findings of Parker *et al.* (2013), despite a different line of genetic evidence and indeed different choice of echolocating species.

## Supplementary Material

Supporting Information
