# Supplementary material for: Sensory Rewiring in an Echolocator: Genome-Wide Modification of Retinogenic and Auditory Genes in the Bat Myotis davidii
Source: G3 (Bethesda). 2014 Aug 4;4(10):1825–35. doi: 10.1534/g3.114.011262 (PMC4199690; doi:10.1534/g3.114.011262)
Supplement: Supporting Information [file supp_4_10_1825__index.html]

Sensory Rewiring in an Echolocator: Genome-Wide Modification of Retinogenic and Auditory Genes in the Bat Myotis davidii — Sensory Rewiring in an Echolocator: Genome-Wide Modification of Retinogenic and Auditory Genes in the Bat Myotis davidii — Supporting Information 

# Sensory Rewiring in an Echolocator: Genome-Wide Modification of Retinogenic and Auditory Genes in the Bat *Myotis davidii*

## Supporting Information for Hudson *et al.*, 2014

**Files in this Data Supplement:**

- Supporting Information - Files S1-S3 (PDF, 224 KB)
- File S3 - Figure displaying BioGPS normalized tissue expression for the gene TSPAN10, highly biased in *M. lucifugus*. (PDF, 436 KB)
- File S1 - CUB analysis for *M. davidii* and *P. alecto*. (.xlsx, 5 MB)
- File S2 - CUB analysis for *M. lucifugus* and *P. vampyrus*. (.xlsx, 2 MB)
