# Supplementary material for: Sensory Rewiring in an Echolocator: Genome-Wide Modification of Retinogenic and Auditory Genes in the Bat Myotis davidii
Source: G3 (Bethesda). 2014 Aug 4;4(10):1825–35. doi: 10.1534/g3.114.011262 (PMC4199690; doi:10.1534/g3.114.011262)

G3·Genes | Genomes | Genetics

SENSORY REWIRING IN AN ECHOLOCATOR: GENOME-WIDE MODIFICATION OF RETINOGENIC AND AUDITORY GENES IN THE BAT *MYOTIS DAVIDII*

Nicholas J. Hudson[1*], Baker ML[2], Hart NS[3], Wynne JW[2], Gu Q[4,5], Huang Z[6], Zhang G[6], Ingham AB[1], Wang L-F[2,7] and Reverter A[1]

[1] Computational and Systems Biology, CSIRO Agriculture Flagship, Queensland Bioscience Precinct, 306 Carmody Road, Brisbane, Queensland, Australia

[2] Australian Animal Health Laboratory, CSIRO Biosecurity Flagship, Geelong, Victoria, Australia

[3] School of Animal Biology and the Oceans Institute, University of Western Australia, Crawley, WA 6009, Western Australia, Australia

[4] College of Information Sciences and Technology, Donghua University, Shanghai 201620, China

[5] Faculty of Medicine, Imperial College London, South Kensington, London SW7 2AZ, England

[6] BGI-Shenzhen, Shenzhen, 518083, China

[7] Program in Emerging Infectious Diseases, Duke-NUS Graduate Medical School, Singapore

*Author for correspondence

Dr. Nicholas James Hudson

nick.hudson@csiro.au

DOI: 10.1534/g3.114.011262

Files S1-S2

Available for download as Excel files at http://www.g3journal.org/lookup/suppl/doi:10.1534/g3.114.011262/-/DC1

File S1 CUB analysis for *M. davidii* and *P. alecto*

File S2 CUB analysis for *M. lucifugus* and *P. vampyrus*

File S3

Figure displaying BioGPS normalized tissue expression for the gene TSPAN10, highly biased in *M. lucifugus*.

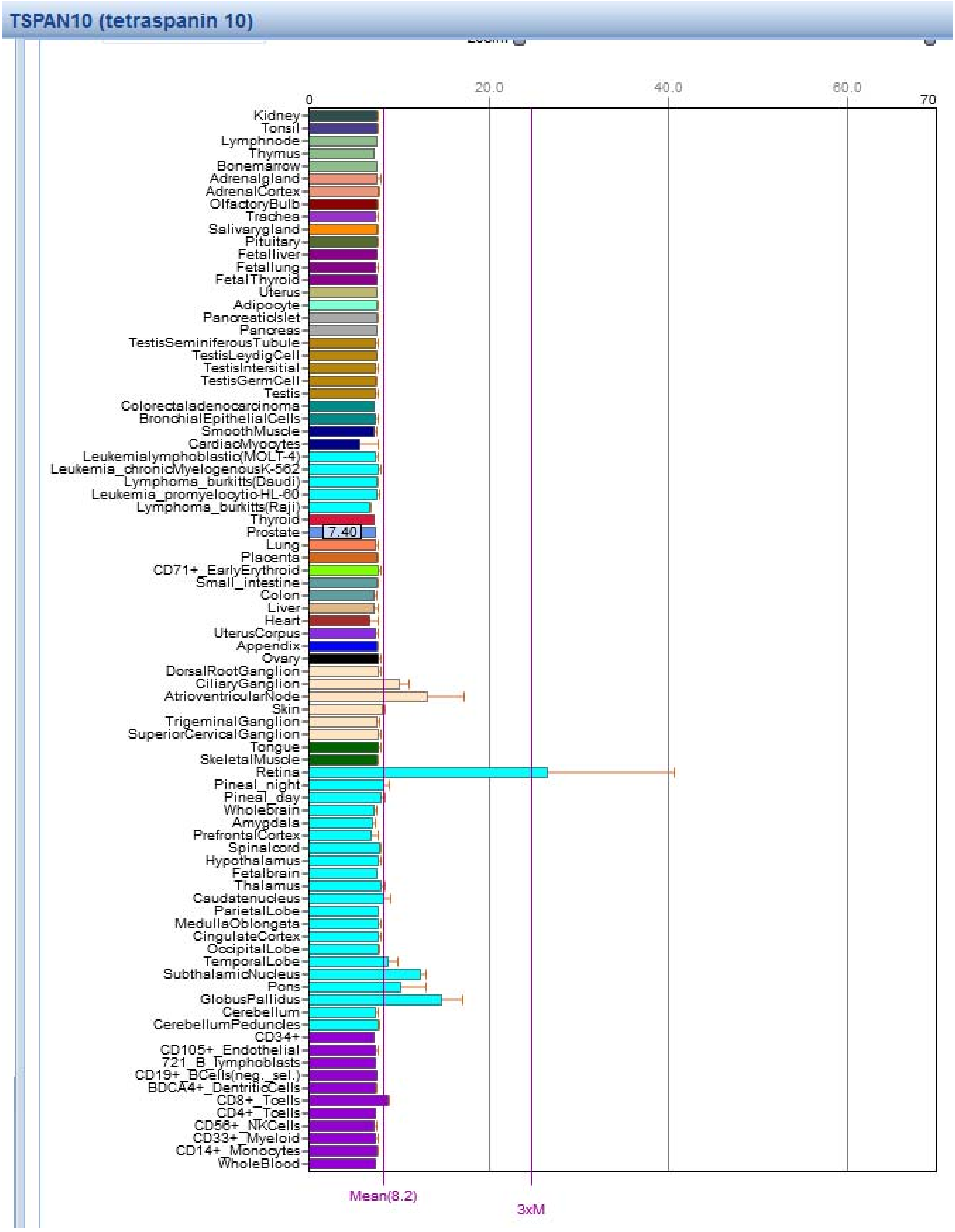

Supplement: Supporting Information [file supp_g3.114.011262_011262SI.pdf]
