# Supplementary material for: Sensory Rewiring in an Echolocator: Genome-Wide Modification of Retinogenic and Auditory Genes in the Bat Myotis davidii
Source: G3 (Bethesda). 2014 Aug 4;4(10):1825–35. doi: 10.1534/g3.114.011262 (PMC4199690; doi:10.1534/g3.114.011262)

File S3

Figure displaying BioGPS normalized tissue expression for the gene TSPAN10, highly biased in *M. lucifugus*.

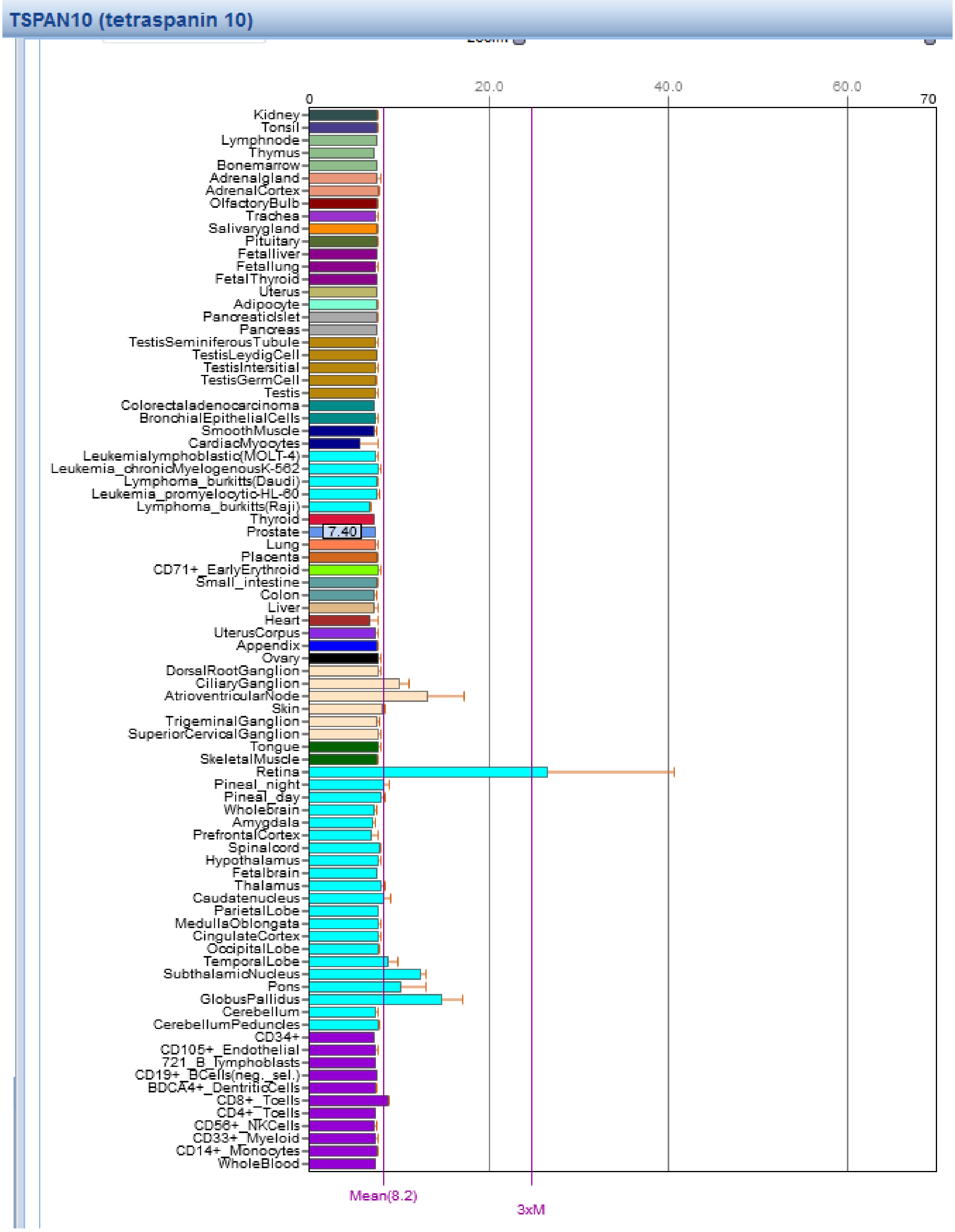

Supplement: Supporting Information [file supp_g3.114.011262_FileS3.pdf]
